# Neophobia, sensory experience and child’s schemata contribute to food choices

**DOI:** 10.1007/s40519-024-01657-5

**Published:** 2024-04-08

**Authors:** Viviana Finistrella, Nicoletta Gianni, Danilo Fintini, Deny Menghini, Silvia Amendola, Lorenzo Maria Donini, Melania Manco

**Affiliations:** 1 Unit of Predictive and Preventive Medicine, Bambino Gesù Children’s Hospital, IRCCS, Via F. Baldelli 38, 00146 Rome, Italy; 2 Endocrinology and Diabetology Unit, Bambino Gesù Children’s Hospital, IRCCS, Rome, Italy; 3 Child and Adolescent Neuropsychiatry Unit, Department of Neurological and Psychiatric Science, Bambino Gesù Children’s Hospital, IRCCS, 00165 Rome, Italy; 4 Experimental Medicine Department, Sapienza University of Rome, Rome, Italy

**Keywords:** Food neophobia, Food consumption, Genetic, Parental control, Weight Status

## Abstract

**Purpose:**

The aim of the present review is to analyze dynamic interactions between nutrigenomics, environmental cues, and parental influence, which can all lead to children’s neophobic reactions and its persistence in time.

**Methods:**

We reviewed studies available on electronic databases, conducted on children aged from birth to 18 years. We also considered official websites of Italian Institutions, providing advice on healthy eating during infancy.

**Results:**

Modern day societies are faced with an eating paradox, which has severe and ever-growing implications for health. In face of a wider availability of healthy foods, individuals instead often choose processed foods high in fat, salt and sugar content. Economic reasons surely influence consumers’ access to foods. However, there is mounting evidence that food choices depend on the interplay between social learning and genetic predispositions (e.g., individual eating traits and food schemata). Neophobia, the behavioral avoidance of new foods, represents an interesting trait, which can significantly influence children’s food refusal. Early sensory experiences and negative cognitive schemata, in the context of primary caregiver–child interactions, importantly contribute to the priming of children’s food rejection.

**Conclusions:**

As neophobia strongly affects consumption of healthy foods, it will be relevant to rule definitively out its role in the genesis of maladaptive food choices and weight status in longitudinal studies tracking to adulthood and, in meanwhile, implement early in life effective social learning strategies, to reduce long-term effects of neophobia on dietary patterns and weight status.

**Level of evidence:**

Level II, controlled trials without randomization.

## Introduction

The developmental process of the child is characterized by a sequence of crucial stages in which growth and development occur [1]. In the early years of life, the brain experiences significant maturation processes, mainly related to the strengthening of communication networks between its constituent nerve cells. Infancy, therefore, represents a phase of extraordinary biological plasticity, facilitating the progressive assimilation of psychomotor, emotional and cognitive skills, which are closely influenced by the surrounding environment.

A crucial element during growth is the consolidation of eating behavior. At this stage it becomes essential to intervene with playful approaches to educate the child to increase food consumption, preventing the risk of malnutrition, either by excess or deficiency. Children may tend to exclude fruits and vegetables from their diets because of color aversions, favoring refined, high-calorie-density foods. This behavior can result in deficiencies of essential micronutrients, such as vitamins and minerals, on the one hand, and in high consumption of carbohydrate-rich foods that may lead to overweight or obesity, on the other. Therefore, it is crucial to adopt educational strategies aimed at promoting a balanced and healthy diet from early childhood. During this growth phase, the child adapts his or her behavior in response to personal experiences, while perceptual skills towards visual, sound and taste stimuli are consolidated and refined. Achievements include the mastery of upright posture, the acquisition of the ability to walk, and the ability to communicate through facial expressions, gestures, and language. Food choices should normally include a wide range of different food items, all necessary to improve healthy lifestyle [1].

However, in recent years, large-scale rapid economic and social transitions have affected health and nutrition patterns of countries worldwide. Food choices have become the object of ever-rising scientific interest, given their potential to influence eating styles and quality of life in children and adults. Indeed, child weight gain seems to result from, among other contributors, inadequate dietary behaviors, and food choices [2] in a time of life when the child starts to taste new foods and learns to enjoy a varied diet. In this light, a decrease in the consumption of fruit and vegetables [3–5] and, potentially, an increase in the consumption of processed foods, especially low-nutrient and energy-dense foods, are considered pivotal [6, 7]. Associations between children’s food consumption, body weight and obesity have been extensively studied. Research has focused on the behavioral determinants that increase the risk for obesity, such as those that shape preferences (i.e., for high fat and sweetened foods) and the rejective behaviors that prevent children from consuming a healthy diet (i.e., fruit and vegetables) [8]. Among these factors, food neophobia (FN) is one of the most important at influencing children’s refusal towards specific food items as well as whole categories of food.

FN can be defined as a behavioral unwillingness and/or fear to sample unfamiliar foods [9] and has been positively associated with children’s avoidance of fruits and vegetables [9–15], as evident from Cooke et al.’s study [16]. It can be distinguished by picky eating, as the last one represents a child refusing a wide range of foods, both familial and unfamiliar [9, 13, 17].

### A modern eating pattern paradox: nutrition transition and dietary acculturation

Over the last three decades, new dietary patterns have spread among communities all over the world as the result of the globalization of the food industry [18]. The mechanization of farm activity has led to an enormous increase in food production but not necessarily to a better variety of foods, with a considerable impact on availability and individuals’ diets. As direct result of these global changes, two strikingly phenomena have taken place. First, we have faced the *nutrition transition*, an accelerated and, sometimes, radical, shift in dietary patterns of individuals from developing countries [19]. As their economies rapidly grow, so does the demand for westernized foods. As a result, traditional diets featuring grains and vegetables are giving way to meals high in fat and sugar and sweetened drinks resulting in a dietary transition, i.e., a major shift from the least developed countries’ predominant underweight problem to the accelerated rise of obesity in developing countries. Recent studies [20–22] investigated dietary pattern among low-income population in Brazil. They found out that eating habits of preschoolers were likely to follow the dietary global trend of the most industrialized countries, characterized by more and more consumption of processed foods. Anjos et al., in 2021 [23], conducted research among Brazilian preschoolers with low socio-economic level, to better address prevention strategies against malnutrition, also in developing countries. They examined FN and food consumption using Child Food Neophobia Scale (CFNS) and Food Frequency Questionnaire (FFQ), respectively, and collecting data on familial educational level. The results have shown that children who were more neophobic, according to the points on the food neophobia scale, had a reduced exposure to Brazilian traditional items, fresh and minimally processed, in favor of increased consumption of chips, cookies, high-fat, high-sugar and low-fiber foods. Less adherence to traditional dietary pattern can be justified by the taste sensitivity trait of neophobic children, who reject foods depending on their appearance, flavor, smell, and texture [23].

What has been observed in most industrialized countries is that *dietary acculturation* profoundly affects individuals’ nutrition habits, corresponding to a growing global trend of homologation of food choices [19]. The dietary acculturation process makes ethnic minority communities becoming more and more acculturated, adopting eating patterns and dietary behavior of predominant groups [19]. Gradually, they consume less traditional foods, less fruit, rice and beans, in favor of sugar, salt and fat foods, which are more common in the predominant groups [19]. They are cheaper than they used to be, thus responding to the economic demands of low-income populations worldwide. This eating pattern paradox is what modern societies are dramatically facing, which results in excessive weight gain and a diet, which is deficient in essential micro-nutrients and fibers, despite a wider food availability [24].

Since that availability and accessibility to foods correlates with the onset of FN in early infancy [23], the easy access to highly processed foods, specifically designed to be palatable, promotes unhealthy eating pattern. Children are increasingly exposed to such foods, instead of less-processed ones; this leading to develop unhealthy preferences and more likely reject those which are healthier. The promotion of these foods in the marketing world, makes them the most advertised ones and makes it difficult to offer unfamiliar foods. Moreover, the high content in sugar, fat and salt accustoms children to a kind of flavor which cannot be encountered in non-processed foods. Hence, while more types of food, including healthy foods, are nowadays readily available in the supermarkets, this availability is not matched by the consumption of a varied range of foods [3, 4].

In the present study, we aim at discussing old studies in the light of the recent ones in a narrative review of the literature, querying the neophobic trait in children and the factors involved in its genesis and persistence in time. By primarily giving its definition and reporting its features and prevalence, our very first purpose is to analyze the multifactorial development of FN in children. We aim at describing genetic, environmental, and social cues and their influence on FN. Secondary outcome is about describing how it can influence health (e.g., body weight) and what strategies can be applied to overcome FN and its implication in ordinary life.

## Methods

**Fig. 1:**
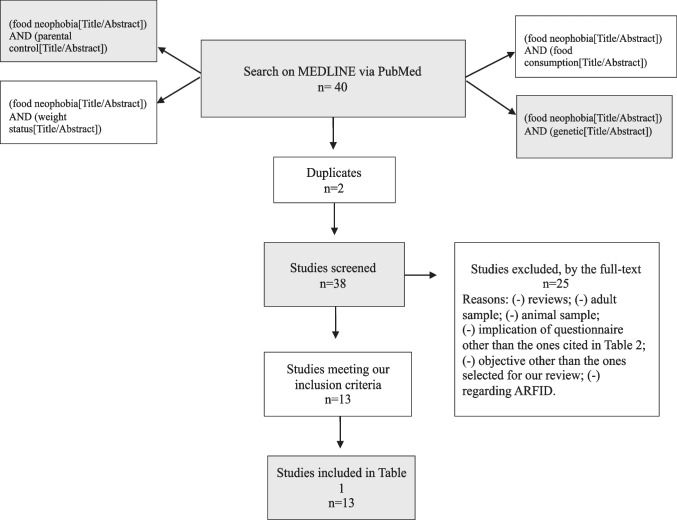
Flow diagram detailing the search strategy

A large body of literature has so far addressed children’s neophobia. In our narrative review, we primarily conducted the search on MEDLINE via PubMed with the following keywords: Food Neophobia, Food Consumption, Genetic, Parental Control, Weight Status, according to the main topics of the present review. As search strategy, while searching, we selected the query Title/Abstract and the abovementioned keywords. As inclusion criteria, we included only cross-sectional and longitudinal studies, applying no restriction for time of publication. As exclusion criteria, studies taking into account only picky eating/food selectivity, among eating disorders, were excluded, due to the difference in features of pickiness and FN. Studies discussing about ARFID (Avoidant/Restrictive Food Intake Disorder) were a priori excluded, since it is an eating disorder, in the food fussiness domain, causing more severe impairments than FN and limiting a wider range of food intake. Some studies were conducted on adults, so they were excluded, being different from our purpose. We also applied restriction for the assessment of FN, including only studies that implicated the validated questionnaire for FN, reported in Table 2. We included studies published in English from 2003 and providing results in children and young individuals (aged birth to 18 years). The last literature search was performed on March 23, 2024. Our search produced 13 studies, as reported in Table 1. The flow diagram below (Fig. 1) details our search strategy.

**Table 1:** Results of the literature search

Authors	Design	N	Age	Objective	Materials and methods	Results
Food consumption						
Cooke et al. [15]	Cross-sectional	564 mother–child dyads	2–6 yy	• Impact of on consumption foods	CFNS [25] FFQ	- ↓ FV, meat - No impact on sweet, fatty snack foods, starchy staples or eggs
Cooke et al. [11]	Cross-sectional	109 parent–child dyads	4–5 yy	• Impact of on consumption of FV and meat	CFNS [25] Mealtime observation	- ↓ FV, protein foods and total calories
Finistrella et al. [26]	Cross-sectional	127 mother–child dyads	2–6 yy	• Association between FN and pickiness during the preschool years • Associations between mothers’ and children’s FN and pickiness • Associations of FN and pickiness with maternal feeding practices • Association of FN and pickiness with child’s over weight/obesity	CFNS [25] CFQ [27] FFQ [28]	- ↔ FN-pickiness - No gender differences - ↔ mothers’ and children’s FN and pickiness - ↔ overweight/obesity with FN and pickiness - ↔ mothers’ food consumption (likes and dislikes)/feeding practices (frequency of proposal of unfamiliar foods) and children’s FN and pickiness - No differences between early feeding type (brestfed, formula-fed or mixed) - No correlation between weaning age and FN and pickiness
Appiani et al. [29]	Cross-sectional	147 parent–child dyads	6–13 yy	• Assess tactile sensitivity • Comparison between lingual tactile sensitivity in children and adults • Association between lingual tactile sensitivity, food preference and consumption (depending on different textures) and FN	Lingual tactile sensitivity assessment with Von Frey filaments [30] Gratings orientation test CFTPQ [31] ICFNS [32] FFQ	- Negative ↔ FN and preferences for hard foods - ↔ Lingual tactile sensitivity to the finest Von Frey filament and FN (in the youngest age group), indicating that children with higher levels of FN are more sensitive to oral tactile stimuli
del Campo et al. [33]	Cross-sectional	600 adolescents	11–18 yy	• Association between taste preferences and FN in adolescents • Gender differences in FN	FNS [34] Haedonic Food Scale [35]	- Preferences for sweet, salty, and umami tastes - FN towards unfamiliar bitter - Few gender differences in FN
Kanisoy and Kabaran [36]	Cross-sectional	300 children	8–10 yy	• Correlation between the Diet Inflammatory Index (DII) scores and dietary quality in children, FN and anthropometric measurements	FNS [34] KIDMED [37] FFQ	- ↔ FN and DII - Negative ↔ FN and KIDMED scores - Negative ↔ KIDMED scores and the DII
Genetic						
Cooke et al. [38]	Cross-sectional	5390	8–11 yy	• Association FN and genetic	CFNS [25]	- Heritable trait of FN
Cassells et al. [39]	Cross-sectional	244 mother–infant dyads	24 ± 1 months	• Association between maternal feeding beliefs regarding underweight and undereating and expression of FN in toddlers	IFQ [40] CFQ [41] CFNS [25]	- ↔ mothers’ concerns about undereating/underweight, lower awareness of their infants’ hunger/satiety cues and feeding practices (pressure to eat) and FN at 2 years - ↔ controlling feeding practices (pressure to eat, restriction), and FN at 2 years
Parental Control						
Wardle et al. [42]	Cross-sectional	564 parent–child dyads	2–6 y	• Association of parental style and food rejection	PC Index [43, 44] CFNS [25]	- ↔ parental control and FV intake
Coulthard and Blissett [45]	Cross-sectional	73	2–5 yy	• Association of taste sensitivity and FV intake • Association of mothers’ FV consumption	CFQ [41] Frequency of FV consumption	- ↔ taste/smell sensitivity and FV Intake - Sensitivity ↓ F intake, regardless of their mothers FV consumption
Tsuji et al. [46]	Cross-sectional	323	4–6 yy	• Association of FV intake, sensitivity to bitterness FN in Japanese preschool children	CFNS [25]	- Sensitivity to bitterness and FN ↔ with consumption of vegetables and soy foods
Weight Status						
Lumeng et al. [47]	Cross-sectional	81 mother–child dyads	3–6 yy	• Association between PROP sensitivity and BMI and influence of FN	CFNS [25]	- No influence of FN score on the relationship between PROP sensitivity and BMI
Roßbach et al. [48]	Cross-sectional	166	10–18 yy	• To assess potential determinants of FN and associations with dietary habits	FNS [34]	- ↓ with increasing age - ↓ with increasing duration of breastfeeding - parental FN ↔ with their children’s FN - FN was associated with underweight as well as adiposity

Studies are organized by topics of our narrative review. Results: Key findings (↓: Decrease, ↔ : Correlation)

*N* number of participants, *Age* age range of participants, *Method: CFNS* child food neophobia scale, *CFQ* child feeding questionnaire, *PC Index* parental control index. *FNS*: food neophobia scale, *IFQ* infant feeding questionnaire, *FFQ* food frequency questionnaire

## Results and discussion

### Development of food neophobia across childhood, epidemiology and diagnosis

FN has not been included as such in the DSM-5-TR (Diagnostic and Statistical Manual of Mental Disorders-5-Text Revision) [49] as it is considered a normal behavior that can modulate food avoidant reactions at different times of the individual’s life and not a disorder. Thus, persistent and severe food neophobic reactions, causing disruption to personal and social life, should be included in the clinical domain as a subtype of eating disorder.

**Fig. 2:**
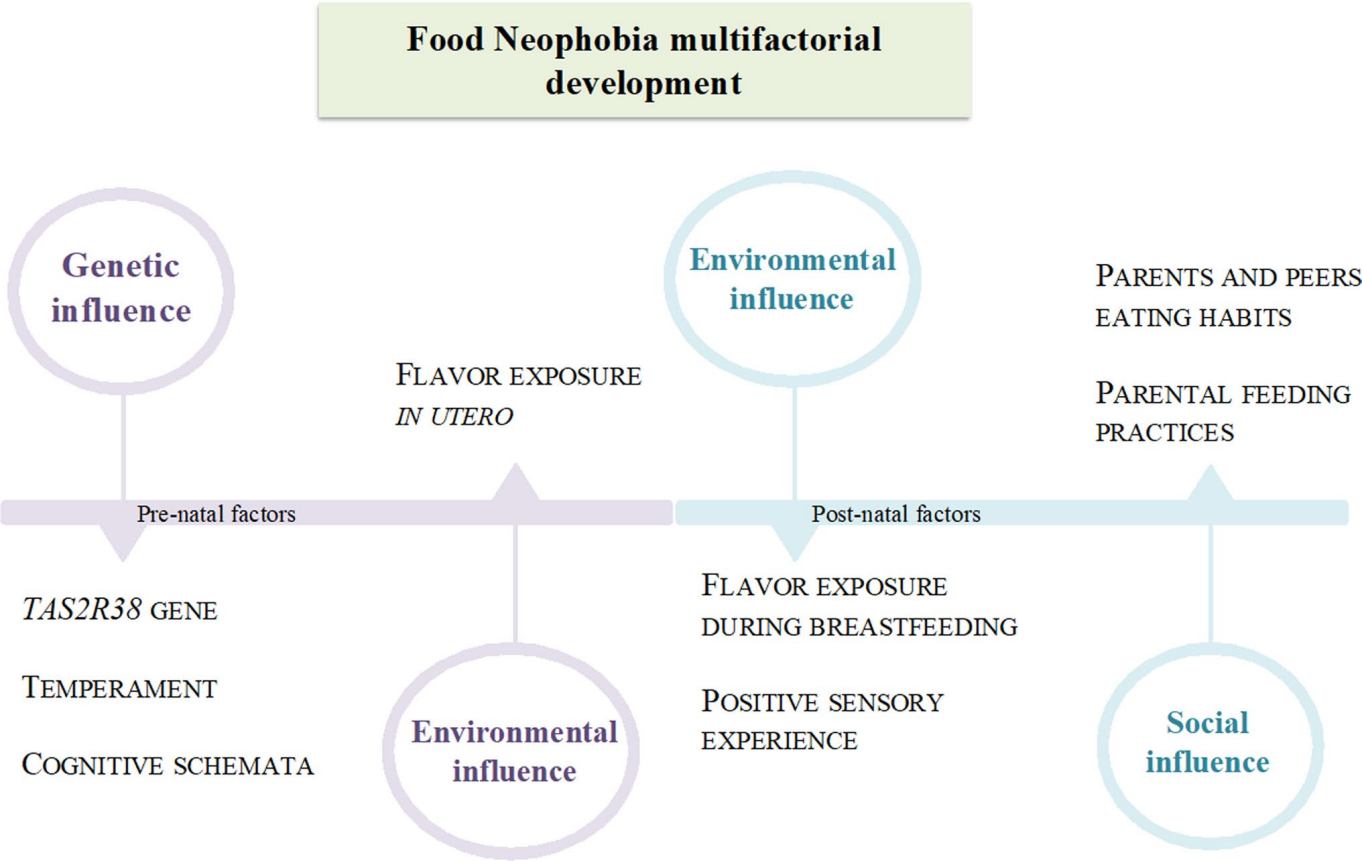
Neophobia’s multifactorial dynamic origin. Neophobia derives from the unique combination of genetic traits, environmental cues and social influence, which are pivotal at different times of the child’s development. Genetic influence [50–53]; Temperament [27, 54]; Cognitive Schemata [55–60]; Flavor exposure in utero [61–65]; Flavor exposure during breastfeeding [66–69]; Positive sensory experience [31, 65, 69, 70]; Parents and peers eating habits [42, 71]; Parental feeding practices [8, 11, 71–79]

Neophobic behaviors derive from a complex interweaving of factors, which become relevant at different stages of the eating behavior development. At birth, genetic predispositions are largely predominant, while, during childhood, social learning influences rejection mechanism through varied and repeated exposures to food in different contexts (Fig. 2).

Characteristically, neophobic behaviors are minimal during infancy, when children usually accept quite readily foods proposed by parents at weaning. Research has brought to light the existence of sensitive period, between 4 and 6 months, in which foods are more easily accepted, as suggested by Mennella and Beauchamp [80], importantly moderating aversive responses. After the first year of life, children often become more alert and start rejecting new foods, refusal behaviors reaching their peak between 18 and 24 months [81]. Other studies report 2–6 years of age as the peak of the FN [61, 72]. As marked, neophobic behaviors appear when children start to walk and are less under parental supervision, this being a genetically determined evolutionary device to prevent toddlers’ ingestion of toxic substances [9, 72, 82]. Noteworthy, Skinner et al.’s longitudinal study [83], on FN and food preferences in 2 to 8 years old children, found that the onset of neophobia early in life was related to the number of foods disliked or never tried at age 8. This suggests the existence of a period when dislike for new foods may become structured and shape later food rejection and choices.

There is strong evidence that FN reaches its peak during toddlerhood and the preschool years and then decreases throughout childhood, as the result of more direct, frequent, and varied experiences with food [9, 61].

It has been estimated that FN can affect around 40–44% of children, in a range age between 4 and 7 years [13, 84, 85], thus severely limiting children’s diet in a time of life when their food options are under construction [86].

FN in children is widely associated to higher consumption of junk food and poorer dietary quality, as demonstrated by Perry et al. [14]. In their study, the authors assessed diet quality in 2-year children, in a sample of 330 parents. They analyzed 3-day 24-h recalls and the CFNS. A higher energy intake from energy-dense food, associated to less consumption of fruit and vegetable, was registered in children who had the highest scores on the CFNS. In addition, Cooke et al. [11], through CFNS and mealtime observation, highlighted a negative correlation between FN and consumption of fruits, vegetables and protein foods. A positive association was found out between Dietary Inflammatory Index (DII) and FN, and a negative one between DII and KIDMED (Mediterranean Diet Quality Index for children and teenagers) scores, when assessing the quality of diet in children, according to a Mediterranean Diet [36].

This notion carries important implications. Even though FN can partially or completely resolve, its consequence for adult food choices is likely to persist. In this light, future research should be able to ascertain the distinctive features of neophobia’s plasticity and adaptability to the environment.

Since 1992 [34], numerous questionnaires have been validated to assess FN in humans. An adjusted scale for children in an age range from 5 to 11 years, the Children’s Food Neophobia Scale (CFNS), was developed in 1994 by Pliner [25], on the model of the previous Food Neophobia Scale (FNS) [34] implicated in adulthood. The CFNS is made up of 10 items and it is administered to caregivers. This instrument has been validated in Canada, and then proposed also in other Countries, such as Portugal [87, 88] and China [89], with just small changes in the items presented. Then, other questionnaires were developed, most of them self-reported by children, such as the Food Situation Questionnaire (FSQ) [90], the Questionnaire on Food Neophobia among French-Speaking Children (QENA) [91], the Fruit and Vegetable Neophobia Instrument (FVNI) [92], the Italian Child Food Neophobia Scale (ICFNS) [32], the Food Neophobia Test Tool (FNTT) [93] and the shortened FNS [34, 93], the Trying New Foods Scale [94] and the Instrument to Identify Food Neophobia in Brazilian Children by Their Caregivers [95]. Since there are differences in availability of foods and cultural eating habits among Countries, some of these questionnaires have been translated and adapted to different Countries, from the original validated tool, as reported in Firme et al.’s review [96]. Here we report a table (Table 2) that synthetize the tools, and their features, developed since 1994 to date.

**Table 2:** Tools specifically developed to assess FN in children

Author	Age	Country	Tool	Items	Interviewee
Pliner [25]	5, 8, 11 yy	Canada	CFNS	10 items	Caregiver
Loewen and Pliner [90]	7–12 yy	Canada	FSQ	10 items and images with facial expressions	Children
Rubio et al. [91]	5–8 yy	France	QENA	13 items and pictures of food	Children
Hollar et al. [92]	8–10 yy	USA	FVNI	18 items	Children
Laureati et al. [32]	6–9 yy	Italy	ICFNS	8 items and images with facial expressions	Children
Damsbo-Svendsen et al. [93]	6–13 yy	Denmark	FNS-6 [34]	6 items	Children
Damsbo-Svendsen et al. [93]	9–13 yy	Denmark	FNTT-6, FNTT-9, FNTT-10	6, 9 and 10 items	Children
Johnson et al. [94]	3–5 yy	USA	The Trying New Foods Scale	9 items and figures	Children
Almeida et al. [95]	4–11 yy	Brazil	Instrument to Identify Food Neophobia in Brazilian Children by Their Caregivers	25 items	Caregivers

Main features from the validation studies. Mod. from: Firme et al. [96]

Other tools investigating eating difficulties in children, also ask some questions regarding FN. They are the Children’s Eating Difficulties Questionnaire [97], An Assessment tool to evaluate the multifaceted characteristics of picky eating habits in children aged 1 to 5 years [98], the Child Food Rejection Scale [99, 100], but their respondents are the caregivers.

### Neophobia versus pickiness and psychological features: personality trait and/or state?

Dietary variety and food choices can be affected by pickiness, a psychological rejection mechanism similar to FN. As such, they may represent “different sides of the same coin” or be distinct behaviors. There is an on-going debate regarding the nature of their relation.

Pickiness applies to children who consistently refuse to eat many familiar food items, their diet being greatly restricted. Wardle suggests that picky and neophobic behaviors belong to the same *food fussiness* domain [101]. However, a study has directly addressed the issue of differential predictors of food neophobia and pickiness in children, even if a correlation between FN and pickiness has been investigated [26]. This study found neophobia to be more linked to genetic predisposition, while pickiness more related to environmental factors [27, 101]. This result is in contrast with a longitudinal study conducted by Mascola et al. [17], which found pickiness to be consistent across development. It showed that, at any given age, between 13 and 22% of the children were reported to be picky eaters, with a 40% of them having a history of pickiness longer than 2 years. Thus, it could be thought of as a relatively stable trait. On the contrary, FN seems to follow different trajectories across lifetime, being both genetically driven but also subject to child development and environmental cues [9].

FN has been hypothesized to be primarily a hereditary trait [86]. In conceptual terms, it can also be defined as a state [102], as it can be modulated by the context (i.e., arousal, flavor response, taste information, and modelling), according to studies conducted by Pliner, Martin, Hobden et al. [103–106].

To date, studies have investigated FN as a one-dimensional construct, without defining a priori whether neophobia was investigated as trait or state. While Pliner et al. [25, 34] were the first to research both dimensions in their studies, however Rigal et al. [102] were the only researchers who proposed a theoretical and methodological distinction between the two dimensions of FN. This distinction may have important implications in terms of incidence, prevalence, and methodology, and should be properly addressed in the near future. Future longitudinal studies could help determine differential origins and possible interaction of these aversive food behaviors, with the purpose of identifying early risk factors and endorsing tailored intervention strategies.

### Cognitive schemata

Information processing relies on models called schemata. A schema is a cognitive framework that helps to organize and interpret information. However, these mental frameworks also cause humans to exclude pertinent information in favor of information that confirms pre-existing beliefs and ideas, making it difficult to retain new information that does not conform to established schemata [55, 56]. Expectations about food stimuli will shape eating behavior and determine not only willingness to try but also post-tasting hedonic response to the food.

For instance, food appearance or taste will strongly influence the first exposure in terms of willingness/unwillingness to try the food item. The subsequent hedonic result will help define the schema both for (1) the specific food (i.e., “*This new food tastes bad*”) and (2) the more general schema for the food category (i.e., “*All novel foods taste bad*”). It has been widely recognized that cognitive schemata about new foods are closely linked to neophobic reactions [57]. Children’s willingness, or conversely reluctance to try, and hedonic response to new food, seems to affect subsequent liking or disliking [58]. In Tuorila and Mustonen’s study [58], neophobic behaviors and reluctance to try a food drove a negative schema and appeared as a strong barrier to further experience.

This sheds new light on the relation between eating behaviors, mental constructs, and genetic background. Cognitive schemata about novel food are commonly more negative than positive [59] and are probably conditioned by genetically based traits such as neophobia, making humans genetically biased towards new foods. Once a schema is formed, it is difficult to change it, probably because of an innate tendency to avoid uncomfortable feelings deriving from conflicting ideas [60]. In the case of a conservative schema, such as a neophobic attitude, a shift to a positive one is brought about only, in time, by a successful repetition of positive food experiences. In keeping with this notion, we take the view that during childhood, dynamic changes in sensory, cognitive, and social development cause individuals to gradually shift their food information processing models. In early childhood, eating experiences are extremely salient for the child, allowing for sensory and regulatory information to be processed bottom-up. These primary experiences define long-term food categorization, both implicit and explicit. In later childhood, a preferential top-down processing of food stimuli takes place, limiting the power of new eating experiences to change behaviors. Future developmental research could explore this schema driven conceptualization by focusing on the important concept of priming in children’s aversive responses.

### Interplay among genetic and environmental determinants

The way how individuals construct their food choices from the earliest ages has become a major issue and, thus, the object of a growing body of literature, with the final purpose of improving modern day children’s diet and preventing the onset of obesity [2]. Food choices develop as the cumulative result of genetics, mental constructs, and environmental cues [86]. Aversive behaviors in humans are usually based on four main reasons:—disgust reaction;—perceived danger related to the food;—inappropriateness of food;—unacceptable combination [107, 108]. Food choices, including preferences and rejection, develop early in childhood and appear to be age dependent, as younger children reject foods more readily on the basis of sensory characteristics, while older children are more aware of danger related to foods [109]; among children in an age range of 4–6 years, sweet taste is the favorite one [110]. When assessing the association of FN and taste preferences, also adolescents aged 11–18 years expressed sweet, salty and umami as the favorite tastes [33]. Food preferences and rejections can importantly predict children’s consumption patterns [111, 112], especially with regard to fruit and vegetable intake [73, 113].

#### Nutrigenomics

Nutrigenomics studies the mutual relationship between foods and genes, aiming at determining “*how food affects a person’s genes and how a person’s genes affect the way the body responds to food*” [114]. The progress of research can lead, in the future, to the development of even more personalized nutrition, basing on individual genetic background, to reduce the risk of diseases [114, 115].

However, FN, as seen above, can be genetically driven. Heritability of neophobia varies according to studies from 67 to 78% in a larger study of 5390 monozygotic and dizygotic twin pairs [38, 116], thus suggesting the strong effect of the genetic inheritance. In Wardle and Cooke’s two-way gene–environment interaction model [86], genes strongly influence the individual’s risk of neophobia, but environmental cues, especially parental ones, up or down modulate gene expression, thus resulting in a graded phenotype of neophobia. Neophobic individuals’ temperament is considered another pointer of its heritability. In fact, they typically present with genetically based temperamental traits, such as emotionality, shyness, sensation seeking, anxiety and neuroticism [27, 54].

Olfactory and gustatory cues are the primary dimensions by which children determine food acceptance. As far as flavor’s importance is concerned in the development of FN, there are scientifically observed inbuilt universal preferences, such as those for sweet tasting foods [117, 118], and some dislikes, such as those for bitter and sour tastes [119]. Therefore, some individuals are genetically prone to detect specific tastes. This is the case of individuals carrying genetic variations in the *TAS2R38* taste gene, which has attracted considerable scientific interest in the past few years. This gene is located on chromosome 7q and three single nucleotide polymorphisms are known (A29P, V262A, I296V), these resulting in two haplotypes: PAV and AVI, identified as tasters and non-tasters, respectively [50]. Carriers are considered propylthiouracil (PROP) bitter tasters or even PROP super-tasters. The latter individuals show a heightened sensitivity to PROP, thus avoiding food rich in PROP [51]. Mennella and Pepino’s interesting studies [52, 53] on the nutrigenomics of taste found that genetic variation of the A49P allele of the *TAS2R38* taste gene influenced bitter perception, being, however, subject to modification by repeated sensory experiences and aging. PROP recognition may be associated with higher prevalence of overweight [47] and may mediate the link between FN and obesity [120, 121]. In fact, evidence showed that taste sensitivity of adolescents with obesity influenced FN. Adolescents with obesity who were PROP tasters were less responsive to weight reduction programs, as they maintained their negative attitude towards fruits and vegetables likely because of the enhanced sensitivity to bitter taste [121]. Such association was confirmed by a subsequent study by Baranowski et al. [122], which found that PROP supertasters had the largest BMI (Body Mass Index) percentile and Z-score. In addition, recent research agrees with these findings. Abaturov and Nikulina [120] conducted an observational case–control study examining 205 children, 6–18 years. They found out that the C/G rs713598 genotype in *TAS2R38* gene is associated with increased risk for metabolically unhealthy obesity. Prepubertal children with obesity and the abovementioned genotype show less to no preference for bitter taste. Thus, the consumption of bitter foods can be potentially replaced with high-fat and sugar ones, contributing to the development of unhealthy obesity. Bitter sensitivity is mainly directed towards vegetables, thus being confirmed by a study aimed at assessing the association of plant-based food intake with sensitivity to bitterness in Japanese preschool children [46]. A higher intake of vegetables characterized the ones having lower scores on CFNS and soy foods consumption was associated with low FN scores in PROP-tasters [46].

Regarding the response to certain tastes, the common dislikes for bitter and sour tastes find their explanation in looking back at biological evolution of human species. These tastes were considered to be potentially toxic as associated to poisonous plants, and to be avoided. Therefore, children are prevented from accidentally ingesting poisonous or toxic substances, by a natural rejection of bitter tasting foods [9, 72, 82, 123]. Nowadays, the risk of ingesting toxic substances through consumption of new foods is minimal, proving that rejection behaviors, which were once adaptive and health protecting, have now become detrimental to the construction of healthy dietary patterns.

#### Sensory experiences

Innate tendencies can be dynamically modified by environmental cues, such as pre- and postnatal experiences. Flavor exposure starts in utero [61, 62]. Between the seventh and the seventeenth weeks of pregnancy the development of gustatory system is concluded, and fetus is exposed via the amniotic fluid to different flavors present in maternal diet, beginning to learn them [63, 65]. Such exposure may dynamically determine phenotypical expression of the individual’s genotype as it happens in the case of sensory perception development [61, 62]. The gustatory system also displays a high degree of neural plasticity from early development, so that sensory systems adapt to changing environmental influences by coordinated alterations in structure and function [124]. Indeed, from the earliest ages, multiple stimulation, involving all senses, contributes to the development of neurosensory pathways. This constant interaction builds differences in response to taste and texture, which, combined, shape the individual’s unique sensory processing style [61, 62]. These early experiences profoundly affect dietary habits as they are acquired soon after exposure and condition later food choices and life-long food habits [64].

Several studies show that early flavor experience and, more importantly, variety during pregnancy or breastfeeding can modify infants’ food acceptance at weaning [65]. Breastfeeding, indeed, exposes children to a wide range of foods, depending on the mother’s dietary pattern [66] and increasing duration of breastfeeding is associated to lower FN scores [48]. During the weaning period, this leads to a minor avoidance of certain flavors, those yet tested. In Mennella et al.’s study [67], babies of mothers who had drunk carrot juice during pregnancy or lactation accepted more readily carrots at weaning. In a later study [68], early food variety was found to be essential in shaping future dietary habits. Babies who had been early exposed to different vegetables, were more prone to accept new flavors than babies exposed only to the single target flavor.

With regard to the development of food choices, during the weaning period, food texture may drive sensory experience, since children respond negatively to certain textures, as demonstrated by infants’ dislike foods with lumps when first introduced at weaning [69]. These sensory features, experienced at the weaning, will influence future eating habits [65]. Texture, by driving likes and dislikes for certain foods, has its impact on the onset of FN [31]. Texture preferences can be related to energy intake, with children eating thicker foods, reducing their energy intake [70, 125]. A tool (CFTPQ, Child Food Texture Preference Questionnaire) has been validated to investigate children texture preferences [31]. By presenting different pairs of foods from different textures (generally named as Hard and Soft), the CFTPQ and ICFNS were administered to European children, aged 9–12 years, and their parents [31]. Findings have shown a higher rate of FN in children who prefer soft foods. Instead, the ones who prefer hard textures seem to have healthier eating pattern, being less neophobic [31]. These children are generally more likely to accept foods with unappealing sensory properties [126]. A study [29] evaluating tactile sensitivity using Von Frey filaments, aimed at assessing the correlation of FN to lingual tactile sensitivity, food preference and consumption, depending on different textures. They concluded that higher score on the ICFNS were negatively associated with preferences for hard foods and children with higher levels of FN were more sensitive to oral tactile stimuli.

Therefore, sensitiveness to all kind of sensory stimuli (also tactile, visual, olfactory and auditory, other to gustatory) links with the liking of softer and more uniform foods. Indeed, neophobia is also characterized by a heightened sensory sensitivity (i.e., how much the child is affected by changes in sensory stimuli) [86] and has found to be a potential characteristic among children with higher rate of sensitiveness [127].

A cross-sectional study [45] has addressed this subject by investigating the correlation between specific sensory processing styles, FN and fruit and vegetable consumption in children. In 7–11 years old children, the refuse to try new vegetable, was associated with FN and the rating of the taste was related to the ratings of olfactory and tactile sensations [128]. When a familial food was presented, the sensory characteristics were rated more positively, than the unifamilial one. Coulthard and Blissett [45] found that preschoolers with gustatory and olfactory sensitivity ate less frequently fruits and vegetables and were less influenced by parental modelling or feeding style.

As these premises highlight the complex nature of the food choice mechanism, we take the view that, as in the case of obesity [129]*,* a developmental perspective is the best way to explain the differential outcomes of variables that contribute to shaping food choices. Sensory development, the child’s developmental traits and parental feeding style jointly contribute [61, 130–132].

Further research should expand existing knowledge, particularly on nutrigenomics, timing of neurosensory pathway development, with the aim of identifying children with sensory sensitivity early in life, when sensory experience with food stimuli has just started, thus avoiding the construction of negative sensory memories.

### Food neophobia and weight status

As seen above, neophobia can constitute a major barrier to the consumption of whole categories of healthy foods, especially fruit and vegetable. Accordingly, it is a strong candidate for substantial interaction with increased, or indeed reduced, body weight. In a systematic review of the literature, Brown et al., in 2016, [133] have reviewed 41 studies; 17 of them did not show any association of FN with body weight. Instead, 6 out of the 41 studies, suggested a possible interaction with underweight, confirming the results of Cooke et al.’s study [16]. However, the most interesting association to be explored is the one that links neophobia to the onset of obesity, highlighted by 2 out of the 41 studies. In fact, the basal genetic predisposition, that guides both the avoidance of unpalatable foods and the sugar–fat–salt preference [134], may contribute to obesity, and strongly counteract efforts to treat it. The relationship between FN and childhood obesity has been also explored in adolescents, in association with taste sensitivity before and after a 1-year residential weight reduction program [121].

These findings bring us to suggest that the relationship between FN and weight status may be mediated by taste sensitivity, but research has focused, with contradictory results, mostly on bitter and sweet perception [135], pointing to the need of a wider investigation on the multiple aspects of sensory development in children with obesity.

In the current obesogenic environment, the challenge that researchers and clinicians must take is how to transform low-energy-dense foods from unlikeable to enjoyable and palatable, as sweets and fatty foods already are. Accordingly, the study of the development of taste receptors, neural relay, and hedonic response, with relation to FN and homeostasis of body weight, must be one of the next important issues to investigate.

In addition to changes in body weight, FN, due to the low consumption of fruits and vegetables, correlates with higher risk of deficits, especially vitamin E, vitamin C, vitamin A, vitamin B6, folate, zinc, iron, calcium, magnesium, fiber [8, 9, 12].

### Food neophobia in children with autism spectrum disorders

Autism spectrum disorder (ASD) is a neurodevelopmental condition that can profoundly affect several aspects of daily life, sometimes being limiting. This disorder is primarily identified by challenges in social and communicative interactions as well as behaviors and interests that appear limited and repetitive. One of the multiple spheres affected includes eating: children with autism may encounter obstacles during mealtimes, due to variables such as individual clinical specificities or the environment in which mealtimes take place. These difficulties can affect not only the child's daily routine but also the family balance, since meals should represent moments of togetherness and sharing.

Numerous research studies highlight that FN is a common problem among people with ASD, persisting into adolescence and suggesting that eating difficulties at an early age may indicate a risk for ASD [136–140]. The DSM-5-TR [49] includes among its diagnostic criteria for ASD the presence of repetitive interests and behaviors that extend to eating, characterized by “*extreme or ritualistic reactions regarding the taste, smell, texture, or appearance of food, or excessive food restriction*” [49]. Feeding difficulties In ASD are characterized by consumption of certain types of food, such as snacks, processed foods, while the major issue is represented by the reluctance towards fruits, vegetables, and proteins [141]. Preferences are often mediated by brands, packaging, the spatial disposition of foods on the plate [142]. These elements significantly complicate meal management for parents in both home and outdoor settings. Crying, screaming, aggressive or escape behavior, distress, spitting, physical rejection, and irritability are often faced in children with ASD and feeding difficulties. Such reactions make caregivers intervening by adapting to the child's needs and altering the menu to accommodate them. This dynamic leads the child to avoid unwanted foods and perpetuate problem behaviors, while the adult learns to prevent such situations during mealtimes by giving in to the child's requests, as pointed out by Ledford and Gast [139].

The prevalence of FN in children presenting with ASD is still a matter of research. Higher levels of neophobia have been found in children with ASD, as compared with children with neurotypical development [143]. De Almeida et al. (2022) [144] have estimated it among Brazilian children: administering a specific and validated questionnaire [95] to caregivers of children aged 4–11 years with ASD, 75% of them resulted in high scores of neophobia.

FN, together with food selectivity, can lead to imbalance intake of nutrients and poor nutritional status, due to monotonousness of food choices [136, 144, 145]. A study conducted by Stafford [145] has found out that the most autistic-type traits the children present with, the higher FN features they have [146]. Moreover, the association of FN and ASD results in high BMI [145, 146]. Even though further investigations are needed, it can be related frequent consumption of energy-dense foods [145, 146]. A link between ASD and excess of body weight has been shown, independently from neophobia, due to tendency to overeat [146–148].

### Role of caregivers

#### Parental eating and feeding styles

Eating behavior develops within different social environments that shape responses to food stimuli. Social learning begins with the primary interactions with caregivers, which are highly significant from birth [149]. Children learn from the caregivers what, how and when to eat. Parental eating attitude, parental feeding styles and food parenting practices influence children’s neophobic behaviors, through modelling [42, 150]. Food parenting practices consist of strategies used by parents to propose new foods to children [71, 72, 74], while parental feeding styles refer to demandingness and responsiveness of parents [79]. They qualify as four different styles: authoritative, authoritarian, indulgent and uninvolved. The authoritative one, referred as high demandingness on child’s eating and high responsiveness to children’s needs, appears to be the most successful one. It leads to a major independence of children in regulating themselves, without coercive practices, since the parents maintain a partial control on children’s eating: they expose them to new foods, but children are allowed to choose what to eat. Indeed, children’s whose parents have an authoritarian feeding style, eat foods high in fats and sugar less frequently, but they did not gain autonomy in choices, due to high demandingness and low responsiveness to children’s wants [79]. Parental control, as well as pressure to eat and mothers’ concerns about undereating and underweight of their children correlate with FN at 2 years and fruits and vegetables intake [39, 42].

Wardle et al. [42] found that parental intake and children's FN are stronger predictors of children's fruit and vegetable consumption than parental control. Parental control could be a behavioral response to children negative attitude to unfamiliar foods. Parental feeding styles, both as a consequence or a cause of children’s refusal to unfamiliar foods, emphasize the importance of the association between parental eating and feeding styles and children’s neophobia and dietary habits [71]. Finistrella et al. [26] found out a correlation between mothers’ food consumption (in terms of likes and dislikes) and children’s FN and pickiness. They also confirmed the association between feeding practices (e.g., frequency of proposal of unfamiliar foods) and FN in their children [26].

Cappellotto and Olsen’s study [127] examined the correlation between parents’ and children’s preferences in terms of food textures, by comparing CFTPQ [31]. In adulthood, harder items are the favorite ones, due to the physiological development of gustatory system, that makes it possible to accept also less-uniform textures. Being exposed to a wide range of foods during the lifetime is hypothesized to be a positive factor in increasing acceptance of variety of textures [127].

It is noteworthy that parents’ food choices have a more prevalent effect on children’s positive attitude towards foods than on food refusal. This suggest that genetically based negative eating behaviors are more difficult to modify by experience and require ad hoc learning strategies [127].

#### Parental feeding practices

Studies have shown that FN and its progression is correlated to parental feeding practices, such as exposure to unfamiliar foods [9]. According to Wardle et al. and Dovey et al. [9, 75], a rejected item should be proposed up to 15 times, in many different textures [12]. If foods are frequently offered to the child, after several exposures they cease to be unfamiliar, and the typically neophobic reactions of fear and disgust can in time be overcome. This means that the child becomes able to recognize the food within a positive schema, in terms of taste, visual representation and category to which the food belongs. Excessive pressure from parents or caregivers in presenting an item to the children or any prompt to eat, together with the common habit of using food as a reward, contribute to the rejection of that foods and to the development of unhealthy eating habits [73, 77].

### Strategies to overcome the food neophobia

Recognition of the role of FN in children’s food refusal can be pivotal to reduce the impact on body weight, both underweight and overweight. Recent research has unveiled strategies aimed at reducing neophobia, that can be of essential use within current nutritional programs directed at changing children’s and families’ dietary habits.

**Fig. 3:**
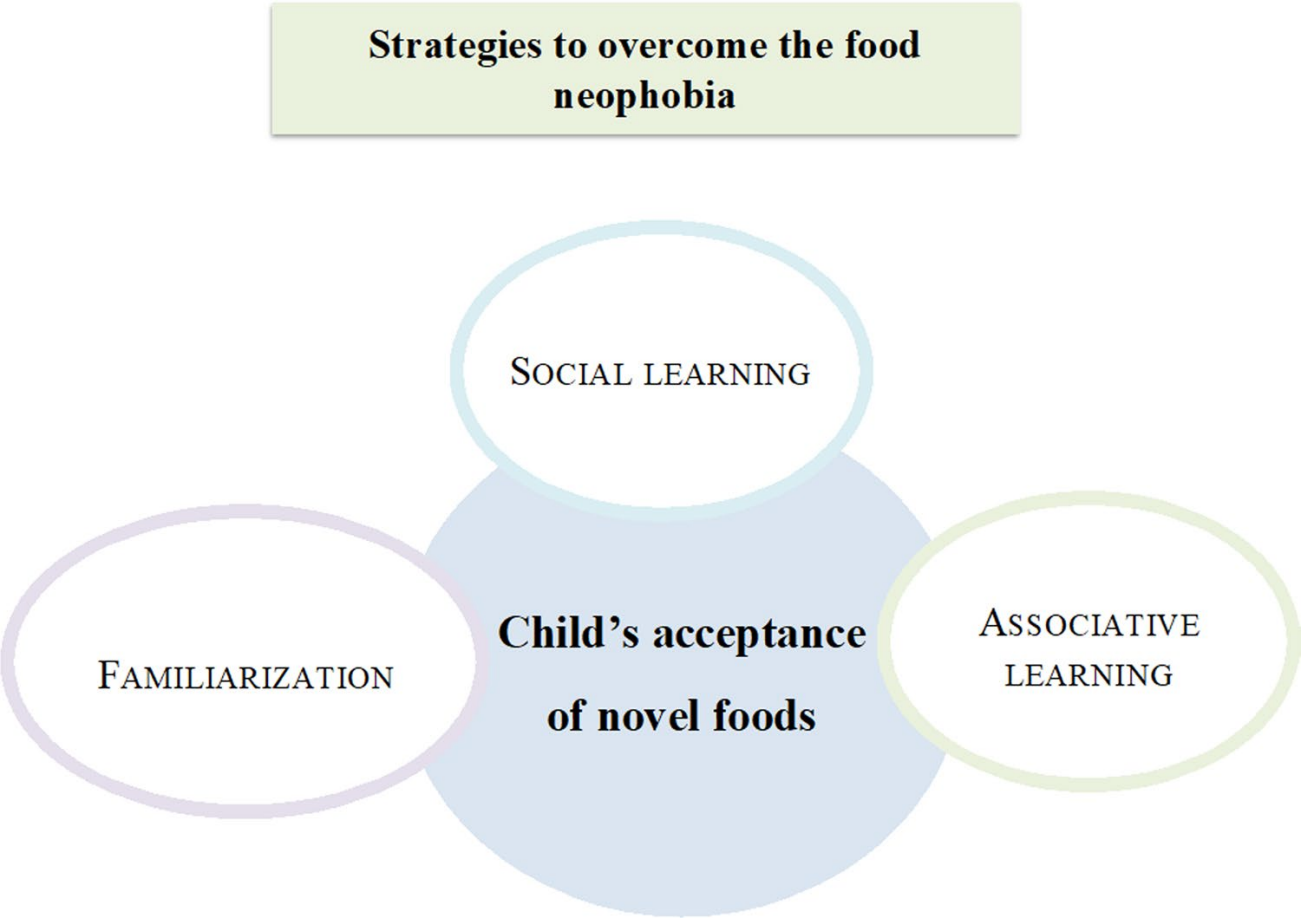
Multilevel strategy that can foster children’s food acceptance of novel/healthy foods. Familiarization, social learning, and associative learning are part of an integrated intervention strategy for the promotion of children’s acceptance of novel and healthy foods. Familiarization [9, 12, 66, 73–78, 151, 152]; Social learning [71, 72, 77, 153]; Associative learning [71, 78, 154]*.*

In this light, strategies that have proved to be effective are based on a multilevel approach (Fig. 3).

#### Familiarization

It consists in discovering food with all senses, touch, taste, smell, but also vision and hearing, creating a positive environment, this allowing the children to build positive memories around that experience [74, 76]. Hence, managing foods is a key-strategy that increases the desire to eat them. Therefore, involving children in meal preparation and portion [78], sensory play and activity based on the use of real foods (e.g., fruits and vegetables) helps them in the process of familiarization and acceptance of foods [151, 155]. Preschoolers playing with real foods were more likely to accept them, as compared to children playing with non-real foods [152]. Engaging neophobic infants with a nutrition education, especially a sensory-based food education program well-structured by professionals and parents, makes the experience with food less frustrating and traumatic and helps them to get into healthy eating habits [66, 78].

#### Social learning

Children learn to like food by modelling their caregivers’ behaviors within a warm and affective eating context. Thus, if children frequently experience seeing their parents and peers while enjoying unfamiliar healthy foods, they learn from their behavior how to approach the foods and acquire a positive model, which can foster their future palate for healthy foods [71, 77]. Addessi et al. [72] examined the acceptance of some foods among children exposed to them in 3 different conditions, while eating: (1) having their parent present, without assuming foods (*Presence* condition); (2) having their parent present, assuming a different item (*Different color* condition); and (3) having their parent present and eating the same food as the child, at the same time (*Same color* condition). As expected, children in the third condition were more prone to accept foods. According to other studies, also expressions of disgust negatively influence willingness to taste them [153].

#### Associative learning

The context and consequences of eating certain foods can make them more palatable to children. This knowledge has been widely used by the food industry but not by families and communities to promote healthy eating [154]. For example, preference for healthy foods can be acquired by the association with a positive environment (bright, warm room colors, appealing presentation, presence of peers or parents to eat with etc.), or with the possibility of enjoying an activity particularly favored by the child [71]. A positive environment was recreated in the study conducted by Kähkönen et al. [78], where a sensory-based food program was proposed to children. It entertained children by visiting farms and forests, singing, drawing, and doing physical activities. During these activities, a buffet-type meal was offered, including several vegetables and fruits. Results demonstrated that the sensory-based food program promoted children’s willingness to try the proposed foods [78]. This happened the most in the group of children whose mothers have low education level, maybe because they daily offer less variety of foods, as compared to highly educated parents [78]. However, the association between sensory-based food program and the rate of FN is still object of research. Strategies that, until now, have been extensively used to condition preference for “junk” foods can and should be used to promote instead children’s acquisition of healthy food choices.

Finally, as reported in a systematic review [66], to adopt the effective strategies presented above, an interplay between healthcare professionals (psychological, nutritional, physical areas) is needed, so that parents can be guided in handling their sons’ neophobia. Since that children often homologate to their parents’ behaviors, promoting healthy lifestyle also in adulthood can be useful, in becoming an exemplum for youngsters.

## Strength and limits

The strength of our study is due to offering a holistic view not only about factors involved in FN onset, but also about the intervention strategies that can be implemented. Indeed, FN represents a very common trait in childhood, possibly compromising weight and eating habits. However, it is often underestimated and simply considered a transitory behavior, happening in different stages of children’s life. Giving advice on a multilevel approach, may help in identifying and treating the most severe FN traits.

We identified the omission of assessment of bias risk as one of the main limitations of our study. We are aware that analyzing risk of bias is pivotal in evaluating the reliability of study results. This may have affected the accuracy of our findings.

## What is already known on this subject?

To date, the role of genetic and social influence on food choices was already assessed. However, the lack of a complete review analyzing the link with modern dietary acculturation and nutrition transition and the need of a study that treats FN in the light of all these perspectives, makes our study new and crucial.

## What this study adds?

The present study offers a comprehensive overview of the literature, resuming factors involved in developing of FN trait in children, all at once, from multiple points of view: nutritional, genetic and environmental ones. Moreover, it considers the pivotal role of caregivers in overcoming the FN behavior and summarizes strategies to reduce its negative impact on lifestyle and dietary habits.

## Conclusions

In westernized countries, unhealthy eating behaviors are spreading faster than ever among children, irrespectively of age, gender, ethnicity and socioeconomic status. FN is one of the key aspects of these maladaptive food choices. In this regard, research has shown that it could be potentially a permanent barrier to children’s acquisition of a positive relationship with healthy foods. However, it can also express itself as a normal phase of children’s eating development with limited effect on subsequent dietary habits. Neophobia’s differential pathways depend on the knowledge researchers and clinicians can acquire on its antecedents, course and interactions and how this knowledge is transformed into effective prevention and treatment strategies. In this light, recent findings strongly point to the existence of period prior to its emergence, when food refusal can be easily avoided, or its effects lessened during the neophobic phase. In the future, the investigation of this specific time frame with relation to children’s sensory, cognitive, and affective schemata may bring invaluable knowledge to the understanding of the development of healthy food choices.

The psychological aspects and consequences of child FN still need to be clarified to reach a consensus for treatment options. Systematic new evidence in these areas may open new perspectives for the successful intervention on maladaptive food choices.

## Data Availability

Not applicable.

## Abbreviations

i.e.: Id Est
FNS: Food neophobia scale
PROP: PROPylthiouracil
BMI: Body mass index
